# Clot Imaging Using Photostable Nanodiamond

**DOI:** 10.3390/nano13060961

**Published:** 2023-03-07

**Authors:** Samuel J. Francis, Marco D. Torelli, Nicholas A. Nunn, Gowthami M. Arepally, Olga A. Shenderova

**Affiliations:** 1 Division of Hematology, Duke University Medical Center, Duke University, Durham, NC 27710, USA; 2 Adámas Nanotechnologies, Inc., Raleigh, NC 27617, USA

**Keywords:** nanodiamond, nanoparticles, fluorescence, imaging, microscopy, thrombus, blood

## Abstract

While thrombosis is the leading cause of morbidity and mortality in the United States, an understanding of its triggers, progression, and response to anticoagulant therapy is lacking. Intravital fluorescence microscopy has advanced the study of thrombus formation by providing targeted, multi-color contrast. However, photodegradation of fluorophores limits the application in longitudinal studies (e.g., clot progression and/or dissolution). Fluorescent nanodiamond (FND) is a fluorophore which utilizes intrinsic fluorescence of chromogenic centers within and protected by the diamond crystalline lattice. Recent developments in diamond processing have allowed for the controlled production of nanodiamonds emitting in green or red. Here, the use of FND to label blood clots and/or clot lysis is demonstrated and compared to commonly used organic fluorophores. Model ex vivo clots were formed with incorporated labeled fibrinogen to allow imaging. FND was shown to match the morphology of organic fluorophore labels absent of photobleaching over time. The addition of tissue plasminogen activator (tPa) allowed visualization of the clot lysis stage, which is vital to studies of both DVT and pulmonary embolism resolution.

## 1. Introduction

Thrombosis is the leading cause of morbidity and mortality in the United States (e.g., acute myocardial infarction, deep venous thrombosis, and pulmonary embolism, among others) [1,2]. Although thrombosis and its complications remain an area of intense research scrutiny, many knowledge gaps exist in the understanding of thrombosis triggers, progression, and/or response to anticoagulant therapy.

In the care of patients with thrombotic conditions, understanding time-dependent changes in clot progression and/or resolution is critical. Residual thrombosis confers heightened morbidity and mortality in both deep vein thrombosis (DVT) and pulmonary embolism (PE). DVT patients with residual thrombosis have increased risk of recurrent DVT and PE and severe post-thrombotic syndrome [3,4,5,6]. Residual thrombosis in the pulmonary bed after a first episode of PE increases risk of recurrent venous thromboembolism and chronic thromboembolic pulmonary hypertension, a progressive lung disorder associated with cardiorespiratory failure [7,8,9]. Given the prevalence of these conditions, there is a need for prognostic tests to determine the individuals at risk for these complications.

Over the past several decades, investigators have increasingly relied on intravital microscopy by optical fluorescence to study mechanisms of thrombus formation [10]. Fluorescence imaging increases sensitivity by providing targeted, multiple-color contrast and improved spatial resolution while obviating exposure to ionizing radiation as in computerized tomography (CT) imaging [11]. This technique has shown great utility for real-time imaging of acute thrombosis [12,13], but has limited application for longitudinal monitoring of clots over extended time periods due to photobleaching. This loss of fluorescent signals from continuous illumination prevents prolonged interrogation of test analytes [14,15,16]. Though alternative dyes with increased photostability have emerged, such as quantum dots, these technologies are prone to oxidation, and the toxicity of such materials raises concerns for both handling and utility in biological assays.

Fluorescent nanodiamonds (FNDs) are intrinsically biocompatible fluorescent labels where fluorescence arises from color centers encapsulated within the diamond lattice, providing truly infinite photostability. Different combinations of nitrogen (N) and lattice vacancies (V) are the basis for color centers in diamonds [17]. Specifically, green emission is provided by NVN (nitrogen-vacancy-nitrogen) centers while red emission is provided by NV (nitrogen-vacancy) centers. Though the red emitting NV center is well studied due to its applicability as an optically addressable sensor, the green NVN center expands the emission profile of fluorescents diamond. While near-micron-size diamond particles have been used to label blood clots in vivo [18,19], there has been limited effort to use sub 200 nm FND to obtain structural information or to investigate applications where photostability can be an issue. In this work, we have adapted bright FNDs with either red/near-infrared or green emission for targeted FND imaging potential through an ex vivo model. In this manuscript, we demonstrate and contrast FND’s differential performance to organic fluorescent labels for imaging clots with respect to photostability, temporal imaging, and resolution.

## 2. Materials and Methods

### 2.1. Preparation of FND

Synthetic type Ib HPHT synthetic nanodiamond particles (Diamond Innovations, UA) with average size 60 nm and 120 nm and initial substitutional nitrogen content of ~100 ppm were used for the production of red and green fluorescent colors in this study. Particles were irradiated with high-energy electrons (3 MeV) to fluences of 5 × 10^18^ e cm^−2^ to generate vacancies, and a subsequent two annealing regimes were used to generate specific color centers in the particles. Red FNDs were prepared by annealing at 850 °C for 2 h under vacuum to form NV centers. Green fluorescent particles were annealed at 1800 °C for 2 min using our recently reported [20] rapid thermal annealing approach [21]. Using synthetic diamond particles as a source of particles with green fluorescence has the benefit of much higher brightness (>6×) in comparison with the natural diamond source that was previously used in the field for production of green fluorescent particles. Nitrogen content and related light absorption is an order of magnitude lower in synthetic diamond in comparison with natural diamond resulting in the increased brightness. Subsequent oxidation using standard procedures well established in the literature was used to remove graphitic carbon and provide a carboxylated (–COOH) terminal surface chemistry on the particles [22]. Particle sizes were assessed by dynamic light scattering (Nano ZS, Malvern, UK).

Avidin particles (either Avidin-FND-(Green) or Avidin-FND-(Red)) were produced by carbodiimide activation and reaction with streptavidin [23]. Briefly, FND was transferred to dry dimethylformamide (DMF) by centrifugation. Sulfo-*N*-hydroxysuccinimide (CovaChem 13405, Loves Park, IL, USA) and N-Ethyl-N′-(3-dimethylaminopropyl)carbodiimide hydrochloride (Sigma-Aldrich 3450, St. Louis, MO, USA) were added to particles at 1 mg/mL with sonication and were activated for 15 min. Particles were washed with DMF, then suspended in sterile deionized water with sonication. To the solution, 200 µg streptavidin (New England Biolabs N7021S) was then added. The solution was allowed to react overnight at 4 °C and then was washed three times with water. Finally, the solution was suspended in standard phosphate-buffered saline with 0.1% bovine serum albumin as a stabilizer at a diamond concentration of 10 mg/mL (1% wt).

### 2.2. Blood Collection and Clot Formation

Whole blood (2 mL) was collected from unmedicated healthy donors into a serum separator tube (Becton, Dickenson, and Company, Franklin Lakes, NJ, USA) containing either 100 µL fibrinogen FITC alone (10mg/mL; Molecular Innovations, Novi, MI, USA) or avidin-biotin mixtures including 100 µL biotin-labeled fibrinogen (40 mg/mL, Molecular Innovations, Novi, MI, USA) with 100 µL of FND-labeled avidin (avidin-FND Red or Green) or 100 µL biotin-labeled fibrinogen with 100 µL phycoerythrin-labeled streptavidin (avidin-PE) (Invitrogen, Thermo Fisher Scientific, Waltham, MA, USA). Tissue plasminogen activator (tPA, Alteplase, Genentech, San Francisco, CA, USA) was added to some PBS containing wells after clot formation as described below. Controls included avidin-FND alone without addition of labeled fibrinogen as well as clot alone.

Serum separator tubes containing whole blood with and without labeled fibrinogen were inverted five times to allow for mixing and placed in a 37 °C dark chamber for 2 h to facilitate clot formation. To remove unincorporated fibrinogen or fluorescent labels, the clot was washed three times in 30 mL of phosphate-buffered saline (PBS; Thermo Fisher Scientific, Waltham, MA, USA) and resuspended in PBS in a tissue culture plate (Thermo Fisher Scientific, Waltham, MA, USA). For clot lysis experiments, 100 µL of tPA (1 mg/mL) was added to wells containing PBS. Clots either in PBS alone or tPA-containing medium were then removed after 1 h of treatment for subsequent epifluorescence microscopy. Clots were gently extracted from the wells and placed on clear slides for imaging.

### 2.3. Microscopy

Widefield imaging was performed on an Olympus IX-71 inverted epifluorescence microscope using a mercury lamp excitation under 10×, 40×, and 100× objectives with the following filter sets (Semrock): Green emitting fluorophores (FITC and NVN containing FND-(green)) were excited by blue excitation (FF01-470/28) with green longpass emission (BLP01-488R). Red emitting fluorophores (PE and NV containing FND-(red)) were excited by green excitation (FF01-517/20) with red longpass emission (BLP02-561R). Images were taken with a CCD color camera fitted to the microscope (AmScope, MT5000-CCD-CK) and processed in FIJI [24].

Scanning electron microscopy (SEM) imaging was performed on the ex vivo clot using standard methods [25]. Briefly, clot was fixed using glutaraldahyde-formaldehyde solution. Serial dehydration of the sample was performed using consecutive washes of 50, 70, 90, 100% ethanol followed by hexamethyldisalizine (Sigma-Aldrich, St. Louis, MO, USA). Sputter coating of sample was followed by immediate SEM imaging, with representative images obtained.

## 3. Results

### 3.1. Macroscopic Appearance of Clot and Composition

**Figure 1:**
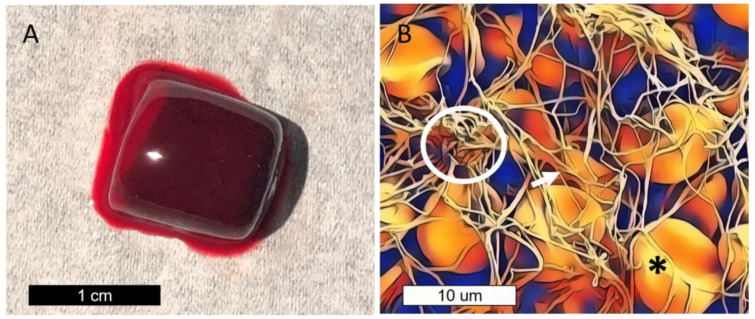
(**A**) Bulk appearance of a clot after ex vivo development in a serum separator tube and subsequent washing. Clots formed are several millimeters in size. (**B**) Colorized scanning electron microscopy (SEM) image of a clot demonstrating the fibrin lattice (arrow), erythrocytes (asterisk), and platelets (circle).

Clotting occurs when fibrinogen is activated by thrombin to form fibrin polymers that enmesh erythrocytes and platelets (Figure 1). In this study, labeled fibrinogen is incorporated into the clot through hemostatic enzymes (thrombin) that activate fibrinogen into fibrin polymers which entrap erythrocytes and platelets into a firm gel. Figure 1A shows the macroscopic appearance of the ex-vivo-formed clot after these steps. The clot gains its red appearance from the high density of trapped erythrocytes (red blood cells). Figure 1B is a colorized scanning electron microscopy (SEM) image of the clot at a microscopic level, showing the fibrin lattice incorporating platelets and erythrocytes into the clot, which provides structural bulk. Optically, the fibrin lattice may be visualized by incorporating fluorescent fibrinogen into the clotting structure. For FND incorporation, biotinylated fibrinogen was used to incorporate labeled fibrinogen into the thrombus for subsequent visualization using avidin-FND.

### 3.2. FND Labeling Is Comparable to Standard Fluorescent Labeling for Imaging Clots

**Figure 2:**
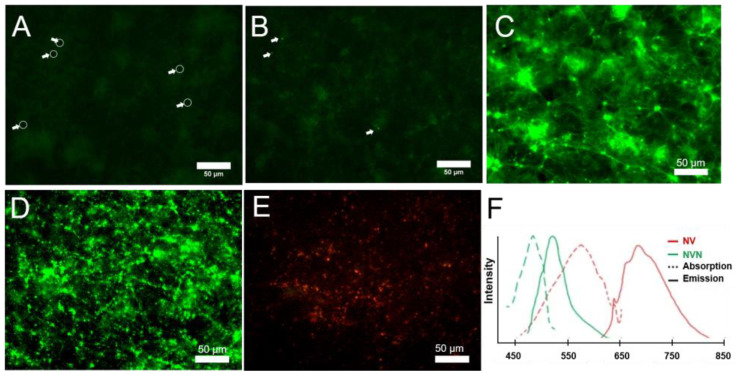
Views of a clot under various conditions. (**A**) Clot alone, with erythrocytes circled and arrowed; (**B**) clot + FND (green) alone (no biotinylated fibrinogen), with individual particles highlighted with arrows; (**C**) clot + fibrinogen FITC; (**D**) clot + biotinylated fibrinogen + avidin-FND (green); (**E**) clot + biotinylated fibrinogen + avidin-FND (red); and (**F**) excitation and emission spectra for NV and NVN containing FND. Scale bars 50 µm. Green Ex/Em: 470/488LP. Red Ex/Em: 517/561LP.

To determine the adequacy of FND to visualize clots, images of the clots needed to be benchmarked to existing commercially available fluorophores and compared to the possible autofluorescence of the blood itself. Epifluorescence microscopy on the clot with no added fluorophore (Figure 2A) with the blue excitation (470 nm) used for the green fluorophores shows the background fluorescence of a clot. There is subtle green background fluorescence without clear organization. This fluorescence arises from oxy-hemoglobin and deoxy-hemoglobin contained within erythrocytes [26] as well as lipids [27]. Non-fluorescing outlines of erythrocytes are also discernable. To evaluate the appearance of a clot with unlabeled FND added to whole blood, clots were formed in the presence of unmodified FND (green) (Figure 2B). While there are some particles visible, seen as individual particles (or small clusters), they are present in a low amount and with no appreciable pattern or contrast present. Figure 2C shows a clot labeled with the common biological fluorophore, FITC, visible under blue excitation. The lattice framework of fibrin and nodes (e.g., due to fibrinogen-bound platelets) is clearly demarcated in this sample, with fluorescence from the FITC fluorophore far brighter than the background fluorescence of panels 2A and 2B. Moreover, the structure resembles examples given in the literature [28]. Figure 2D shows clot labeling via 120 nm avidin-FND (Green), containing NVN fluorescent centers, bound to biotinylated fibrinogen under blue excitation. The same node-like structure is again visualized, with a similar lattice-like pattern seen as with fibrinogen FITC. Of note, linear fibrin strands were not captured in the same manner as with FITC. Finally, 120 nm avidin-FND (red) containing NV centers bound to biotinylated fibrinogen within a clot is shown in Figure 2E under green excitation. Due to the NV centers, these FNDs fluoresce red when undergoing blue excitation. Though the NV center FND image appears dimmer when compared to the NVN, in this set-up the quantum yields of the two cannot be directly compared due to the different filter sets, which have different intrinsic excitation intensities to the mercury arc lamp profile and a non-uniform bias in wavelength quantum efficiency present in the sensor in our CCD (Sony ICX282AQ sensor), which has better green sensitivity. The relative brightness of the centers is similar, as has been quantitatively assessed [21].

### 3.3. Comparison of Particle Size for Labeling: 60 nm vs. 120 nm

**Figure 3:**
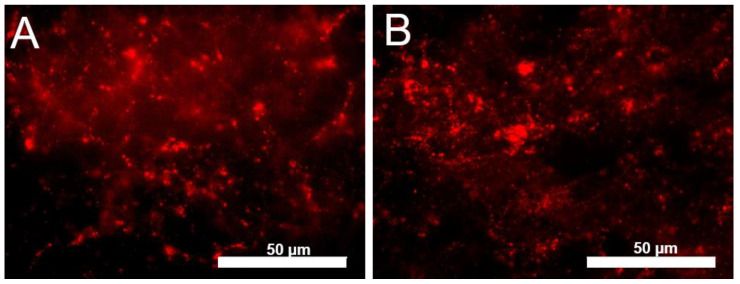
Avidin FND (red) containing NV centers and biotinylated fibrinogen viewed at 100× under blue excitation in formed clots. (**A**) 60 nm FND, (**B**) 120 nm FND. Scale bar 50 µm. Ex/Em: 517/561LP.

For structure labeling, smaller-sized particles could have less steric hindrance in a developing clot and could increase image resolution. However, FND brightness is proportional to particle size, thus smaller particles will be dimmer. To assess these varying effects of smaller FND on clot imaging, two samples of avidin-FND (red) were used for labeling, varying only by size (60 nm or 120 nm) to determine if there was any resolution or fluorescence advantage of one of the sizes over the other. Figure 3 compares clot labeling by 60 nm (33A) and 120 nm (3B) avidin-FND (red) bound to biotinylated fibrinogen. In both cases, the lattice-like pattern of fibrinogen binding is evident. In comparing the two sizes, we noted that the fidelity between the two FND sizes appears similar overall. While there is the potentiality to visualize better features using the 60 nm particles (3A), the image also has a slightly more non-structural haze/background from the NV particles than 3B. In this study, the mass of diamond was kept constant, which results in an ~8× concentration increase. Thus, an optimal amount for labeling should still be defined based on particle size.

### 3.4. FND Bound to Fibrinogen Demonstrate Changes in Clot Exposed to tPA

**Figure 4:**
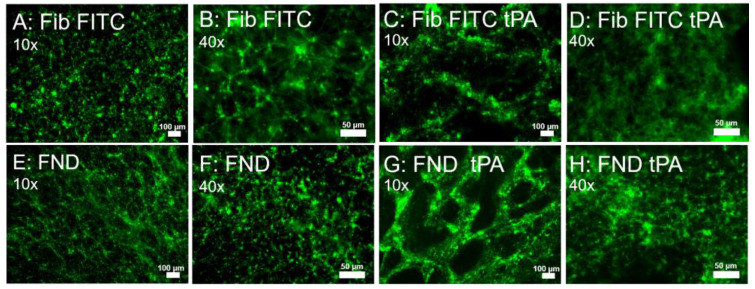
Epifluorescence microscopy examining fibrinogen labeled with FITC or FND in clot 1 h after formation before and after exposure to tPA: Clot containing fibrinogen-FITC without tPA exposure (**A**,**B**) and with tPA exposure (**C**,**D**) at magnifications (10× and 40×, respectively). Clot containing fibrinogen-bio with FND (green NVN)-avidin without tPA exposure (**E**,**F**) and with tPA exposure (**G**,**H**) at magnifications 10× and 40×, respectively. Scale bars are 100 µm for 10× and 50 µm for 40×. Ex/Em: 470/488LP.

In vivo, clots are broken down gradually through fibrinolysis, a process initiated by tissue plasminogen activator (tPA). To examine the utility in monitoring the structural changes of clots undergoing fibrinolysis, our ex vivo clots labeled with avidin-FND or FITC fibrinogen were subjected to tPA treatment. Figure 4A,B show fibrinogen FITC within clots prior to exposure to tPA at two different magnifications. As seen with prior experiments, the lattice structure of fibrin is seen at both magnifications. Figure 4C,D then demonstrate fibrinogen FITC after exposure to tPA for 1 h. There is a clear disruption in the fibrin network within the clot, with the creation of large gaps within the clot highlighting locations where fibrin has been cleaved. Fibrin is still, however, visible within the clot, binding the fibrinogen FITC. Figure 4E,F show 120 nm avidin-FND (green)-labeled clots after 1 h. As seen in imaging at the initial time point, the lattice pattern of fibrin within the clots is visible. The long-range webbed network is especially appreciable at the lowest magnification (Figure 4E). Exposure to tPa, however, induces large structural changes (Figure 4G,H). There are large gaps within the fibrin lattice where fibrin has dissolved. Other areas of the clot demonstrate preservation of fibrin where FND binding is still present. Notably, both the large holes (Figure 4C,G) and the smaller disordered holes (Figure 4D,H) that appear after tPA addition are more readily visualized in the avidin-FND-labeled sample.

### 3.5. FND Eliminates Photobleaching Effects

**Figure 5:**
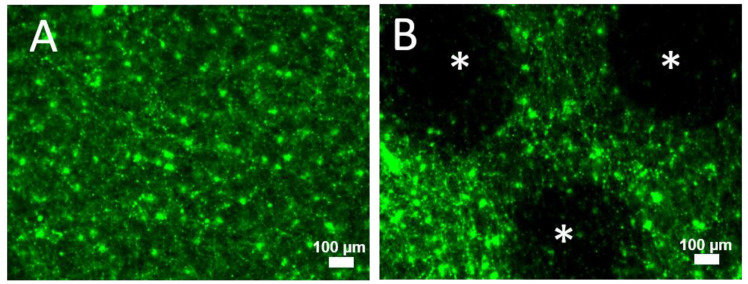
(**A**) Image of FITC-labeled clot at 10× prior to investigation at higher magnification. (**B**) Image of FITC-labeled clot after viewing several areas at higher magnification (40×) and then reimaging at 10×, showing the appearance of “holes” formed due to FITC bleaching (stars). Scale bars 100 µm. Ex/Em: 470/488LP.

Photobleaching is a major limitation of current fluorophores used in imaging applications but does not occur with FND. A practical example is highlighted in Figure 5. Figure 5A shows the appearance of FITC-labeled fibrinogen in the clot freshly imaged at 10× magnification. The area was then investigated in several regions at a higher magnification (40×). In reimaging again at the lower 10× magnification, dark spots were visible that were not originally present. While the phenomena of photodegradation itself is not new, the visual similarity to an induced clot lysis response (Figure 4D) is notable. Specifically, it shows the limitations of imaging the same sample at multiple timepoints during the dissolution process using FITC labeling.

**Figure 6:**
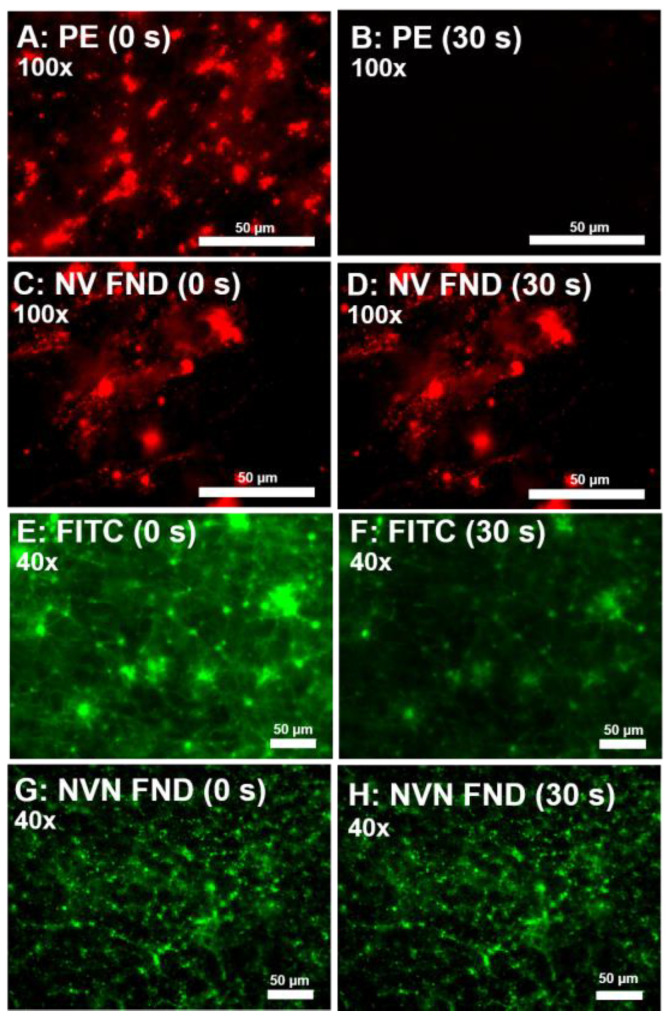
Comparison of photobleaching under different labeling conditions for organic fluorophores as compared to FND fluorophores for red and green emission. (**A**–**D**) Avidin fluorophores labeling biotinylated CD41 at 100×, labeling activated platelets within the clot. Phycoerythrin-avidin-labeled activated platelets are not visible after 30 s (**A**,**B**), whereas FND (red NV) does not change (**C**,**D**). E-H show labelled fibrinogen at 40×. FITC shows dramatic reduction in intensity over 30 s (**E**,**F**), whereas FND (green NVN) does not discernably change (**G**,**H**). Green Ex/Em: 470/488LP; red Ex/Em: 517/561LP.

To compare photobleaching effects, in a separate experiment we compared avidin-FND to the commonly used fluorophores FITC and phycoerythrin (PE), such that both the green and red diamonds could be compared. Imaging conditions were the same for the FND and organic fluorophore in their respective channels (green vs. red). Both PE (Figure 6A,B) and FITC (Figure 6E,F) degrade dramatically over time. After 30 s of illumination from the filtered light of a mercury lamp in a typical fluorescent microscope, PE is almost completely non-visible, dropping by 90%, while the FITC luminescence intensity is dramatically reduced, dropping by 57%. While time for photobleaching decreases at higher levels of magnification for both PE and FITC, due to the fluorophores being exposed to a higher flux of photons, the respective decreases in PE and FITC are consistent with similar studies [16]. Regardless of the time viewed, fluorescence from FND (red) (Figure 6C,D) and FND (green) (6G,H) does not show any measurable change.

## 4. Discussion

FND imaging of whole blood clots provides several potential advantages over conventional fluorophores used in biological imaging. As demonstrated in this series of experiments, FND can be conjugated to biological targets via the avidin–biotin bond. In this environment, their lack of photobleaching allows for temporal imaging and quantification within whole blood and clots. This stability extends to how the reagents are handled compared to other fluorescent materials: quantum dots, for example, are subject to photooxidation which influences surface functionality and emission properties [29]. This characteristic limits their use to environments that are oxygen- and moisture-free [30]. FNDs are able to withstand sterilization through autoclaving, heating, high energy irradiation, and freezing during storage [31,32,33]. Further, the inert core allows for high biocompatibility and low toxicity when compared to others [31,33,34,35,36,37].

Organic fluorophores still achieve better labeling resolution as compared to that of 100 nm particles. Two factors can contribute to this. First, due to their size, particles are sterically less able to label small analytes. Given that fibrils in the clot network are under 100 nm in size [38], 120 nm particles may be unable to label the fine details of these structures. This result is alluded to in Figure 3, but warrants further study. Indeed, the smallest threadlike projections were not as readily captured by FND compared to FITC. Secondly, the fluorescence of the individual FND is dependent on its size for a given color center density. Larger particles have more color centers and thus are brighter. Particle fluorophore FNDs of a given size actually exist as a distribution of sizes, with a corresponding distribution of brightness. For the generation of particles used in this study, the size variance can be up to 15%. Due to the volumetric dependence of fluorescence, this corresponds to a potential brightness difference of up to 50%. This results in the possibility of altered fidelity for each labeled structure because it is possible to have variability in the brightness of uniformly labeled objects.

Despite these limitations of particle fluorophores, their utility for imaging to reveal all essential structural features of the clot was demonstrated. The small size and ability to easily conjugate FND to avidin allows for targeted binding of many antigens within whole blood and the clots that develop. FND is still small enough to allow for penetration and binding to the biomarkers as the clot forms. As FND sizes are ~3% the diameter of platelets and ~1.6% the diameter of erythrocytes, these structures are still readily labeled. Future studies can examine the use of avidin FND (NV) linked to a biotinylated antibody with other avidin FND (NVN) linked to another antibody. This will allow for simultaneous viewing of two separate antigens within a clot over time.

A key advantage demonstrated in these experiments is that FNDs do not photobleach. Conventional fluorophores are limited in their ability to be examined serially within biological tissue by their rapid photobleaching. Both the FITC and PE fluorophores utilized in these experiments began to have photobleaching within seconds of confocal microscopic examination. Moreover, imaging artifacts were noticed during re-imaging in experiments on clot dissolution, when the dark “holes” caused by photobleaching looked similar in appearance to the web-like pattern present in the clot exposed to tPA (Figure 5). FND, on the other hand, has been demonstrated to have no obvious photobleaching over a time course of days [14]. While this leads to practical issues during standard data collection (e.g., must image quickly under minimal illumination conditions), a major strength is the ability to perform subsequent longitudinal studies of thrombosis that have the potential to elucidate the mechanisms by which acute thrombotic conditions can develop sequelae that lead to long-term morbidity and mortality.

Thrombosis and fibrinolysis are dynamic processes in a clot, which often are not routinely examined together in the in vivo setting. A potential application of FND for imaging thrombus formation and dissolution would be clot imaging studies in murine models of thrombosis. In particular, the inferior venous cava (IVC) stenosis model in mice lends itself to such studies, as clots can be monitored over time for several weeks [39]. In this model, the IVC is either narrowed or closed by a silk ligature to decrease blood flow to mimic the reduced flow state that often precedes formation of DVT. This reduced flow is accompanied by complete occlusion with thrombi within two hours of ligation and can be monitored for week afterwards for histologic reactions. Experimental endpoints typically involve sacrificing mice at designated time points and measuring thrombus size, weight, and/or other histologic measurements. This model could be modified for longitudinal imaging of thrombi by injecting mice with platelet- and/or fibrinogen-labeled antibodies containing FND and clots could be imaged over time by immunofluorescence of confocal imaging.

The use of tPA to accelerate fibrinolysis in our experiment demonstrates that FNDs are able to reflect the changes within the structure of the clot as breakdown occurs. We were able to visualize the clot lysis stage, which is vital to studies of both DVT and pulmonary embolism. Being able to quantify temporal changes with individual donor variability may lead to clinical interventions in the future. The ability to better prognosticate those affected by either DVT or PE who are more likely to develop chronic complications could allow for more aggressive management in the acute phase.

## 5. Conclusions

Here, the use of FND for clot imaging was demonstrated for both traditional red (PE) and green (FITC) emission channels using, respectively, NV- and NVN-containing nanodiamonds. Photostability was assessed in comparison to these organic dyes, demonstrating reliable unchanging fluorescence over time. Such a capability is expected to translate to better clinical bedside patient care by allowing for longitudinal assessment of clot lysis in individuals.

## Data Availability

Data available from authors upon request.

## References

[1] Beckman M.G., Hooper W.C., Critchley S.E., Ortel T.L. (2010). Venous thromboembolism: A public health concern. Am. J. Prev. Med..

[2] Pitts S.R.N.R., Xu J., Burt C.W. (2008). National Hospital Ambulatory Medical Care Survey: 2006 emergency department summary. Natl. Health Stat. Rep..

[3] Prandoni P., Lensing A.W., Prins M.H., Pesavento R., Piccioli A., Sartori M.T., Tormene D., Milan M., Vedovetto V., Noventa F. (2015). The impact of residual thrombosis on the long-term outcome of patients with deep venous thrombosis treated with conventional anticoagulation. Semin. Thromb. Hemost..

[4] Kearon C. (2003). Natural history of venous thromboembolism. Circulation.

[5] Huang H., Gu J.P., Shi H.F., Shi W.Y., Lu J.Y., Chen L., Su H.B. (2018). Assessment of the Probability of Post-thrombotic Syndrome in Patients with Lower Extremity Deep Venous Thrombosis. Sci. Rep..

[6] Rabinovich A., Kahn S.R. (2017). The postthrombotic syndrome: Current evidence and future challenges. J. Thromb. Haemost..

[7] Pengo V., Lensing A.W., Prins M.H., Marchiori A., Davidson B.L., Tiozzo F., Albanese P., Biasiolo A., Pegoraro C., Iliceto S. (2004). Incidence of chronic thromboembolic pulmonary hypertension after pulmonary embolism. N. Engl. J. Med..

[8] Kantake M., Tanabe N., Sugiura T., Shigeta A., Yanagawa N., Jujo T., Kawata N., Amano H., Matsuura Y., Nishimura R. (2013). Association of deep vein thrombosis type with clinical phenotype of chronic thromboembolic pulmonary hypertension. Int. J. Cardiol..

[9] Korkmaz A., Ozlu T., Ozsu S., Kazaz Z., Bulbul Y. (2012). Long-term outcomes in acute pulmonary thromboembolism: The incidence of chronic thromboembolic pulmonary hypertension and associated risk factors. Clin. Appl. Thromb. Hemost..

[10] Taqueti V.R., Jaffer F.A. (2013). High-Resolution Molecular Imaging Via Intravital Microscopy: Illuminating Vascular Biology In Vivo. Integr. Biol. Quant. Biosci. Nano Macro.

[11] Wang X., Peter K. (2017). Molecular Imaging of Atherothrombotic DiseasesHighlights: Seeing Is Believing. Arterioscler. Thromb. Vasc. Biol..

[12] Falati S., Gross P., Merrill-Skoloff G., Furie B.C., Furie B. (2002). Real-time in vivo imaging of platelets, tissue factor and fibrin during arterial thrombus formation in the mouse. Nat. Med..

[13] Furie B., Furie B. (2007). In vivo thrombus formation. J. Thromb. Haemost..

[14] Reineck P., Francis A., Orth A., Lau D.W.M., Nixon-Luke R.D.V., Rastogi I.D., Razali W.A.W., Cordina N.M., Parker L.M., Sreenivasan V.K.A. (2016). Brightness and Photostability of Emerging Red and Near-IR Fluorescent Nanomaterials for Bioimaging. Adv. Opt. Mater..

[15] Boisset J.-C., Andrieu-Soler C., Van Cappellen W.A., Clapes T., Robin C. (2011). Ex vivo time-lapse confocal imaging of the mouse embryo aorta. Nat. Protoc..

[16] Tajiri K., Kishi H., Ozawa T., Sugiyama T., Muraguchi A. (2009). SFMAC: A novel method for analyzing multiple parameters on lymphocytes with a single fluorophore in cell-microarray system. Cytom. Part A J. Int. Soc. Adv. Cytom..

[17] Zaitsev A.M. (2001). Optical Properties of Diamond: A Data Handbook.

[18] Marcinkiewicz C., Gerstenhaber J.A., Sternberg M., Lelkes P.I., Feuerstein G. (2017). Bitistatin-functionalized fluorescent nanodiamond particles specifically bind to purified human platelet integrin receptor αIIbβ3 and activated platelets. Int. J. Nanomed..

[19] Gerstenhaber J.A., Barone F.C., Marcinkiewicz C., Li J., Shiloh A.O., Sternberg M., Lelkes P.I., Feuerstein G. (2017). Vascular thrombus imaging in vivo via near-infrared fluorescent nanodiamond particles bioengineered with the disintegrin bitistatin (Part II). Int. J. Nanomed..

[20] Dei Cas L., Zeldin S., Nunn N., Torelli M., Shames A.I., Zaitsev A.M., Shenderova O. (2019). From Fancy Blue to Red: Controlled Production of a Vibrant Color Spectrum of Fluorescent Diamond Particles. Adv. Funct. Mater..

[21] Nunn N., Prabhakar N., Reineck P., Magidson V., Kamiya E., Heinz W.F., Torelli M.D., Rosenholm J., Zaitsev A., Shenderova O. (2019). Brilliant blue, green, yellow, and red fluorescent diamond particles: Synthesis, characterization, and multiplex imaging demonstrations. Nanoscale.

[22] Shenderova O.A., Shames A.I., Nunn N.A., Torelli M.D., Vlasov I., Zaitsev A. (2019). Synthesis, properties, and applications of fluorescent diamond particles. J. Vac. Sci. Technol. B.

[23] Hermanson G.T. (2013). Bioconjugate Techniques.

[24] Schindelin J., Arganda-Carreras I., Frise E., Kaynig V., Longair M., Pietzsch T., Preibisch S., Rueden C., Saalfeld S., Schmid B. (2012). Fiji: An open-source platform for biological-image analysis. Nat. Methods.

[25] Pretorius E., Swanepoel A.C., DeVilliers S., Bester J. (2017). Blood clot parameters: Thromboelastography and scanning electron microscopy in research and clinical practice. Thromb. Res..

[26] Khandelwal S., Saxena R.K. (2007). Age-dependent increase in green autofluorescence of blood erythrocytes. J. Biosci..

[27] Croce A.C., Ferrigno A., Bottiroli G., Vairetti M. (2018). Autofluorescence-based optical biopsy: An effective diagnostic tool in hepatology. Liver Int..

[28] Zamolodchikov D., Strickland S. (2012). Aβ delays fibrin clot lysis by altering fibrin structure and attenuating plasminogen binding to fibrin. Blood.

[29] Van Sark W.G., Frederix P.L., Van den Heuvel D.J., Gerritsen H.C., Bol A.A., Van Lingen J.N., de Mello Donega C., Meijerink A. (2001). Photooxidation and photobleaching of single CdSe/ZnS quantum dots probed by room-temperature time-resolved spectroscopy. J. Phys. Chem. B.

[30] Chashchikhin O., Budyka M. (2017). Photoactivation, photobleaching and photoetching of CdS quantum dots− Role of oxygen and solvent. J. Photochem. Photobiol. A Chem..

[31] Vaijayanthimala V., Cheng P.-Y., Yeh S.-H., Liu K.-K., Hsiao C.-H., Chao J.-I., Chang H.-C. (2012). The long-term stability and biocompatibility of fluorescent nanodiamond as an in vivo contrast agent. Biomaterials.

[32] Faklaris O., Joshi V., Irinopoulou T., Tauc P., Sennour M., Girard H., Gesset C., Arnault J.-C., Thorel A., Boudou J.-P. (2009). Photoluminescent diamond nanoparticles for cell labeling: Study of the uptake mechanism in mammalian cells. ACS Nano.

[33] Yu S.-J., Kang M.-W., Chang H.-C., Chen K.-M., Yu Y.-C. (2005). Bright fluorescent nanodiamonds: No photobleaching and low cytotoxicity. J. Am. Chem. Soc..

[34] Tsoi K.M., Dai Q., Alman B.A., Chan W.C. (2013). Are quantum dots toxic? Exploring the discrepancy between cell culture and animal studies. Acc. Chem. Res..

[35] Pelley J.L., Daar A.S., Saner M.A. (2009). State of academic knowledge on toxicity and biological fate of quantum dots. Toxicol. Sci..

[36] Hardman R. (2006). A toxicologic review of quantum dots: Toxicity depends on physicochemical and environmental factors. Environ. Health Perspect..

[37] Schrand A.M., Hens S.A., Shenderova O.A. (2009). Nanodiamond Particles: Properties and Perspectives for Bioapplications. Crit. Rev. Solid State Mater. Sci..

[38] Yeromonahos C., Polack B., Caton F. (2010). Nanostructure of the fibrin clot. Biophys. J..

[39] Deatrick K.B., Luke C.E., Elfline M.A., Sood V., Baldwin J., Upchurch G.R., Jaffer F.A., Wakefield T.W., Henke P.K. (2013). The effect of matrix metalloproteinase 2 and matrix metalloproteinase 2/9 deletion in experimental post-thrombotic vein wall remodeling. J. Vasc. Surg..

